# E6/E7-P53-POU2F1-CTHRC1 axis promotes cervical cancer metastasis and activates Wnt/PCP pathway

**DOI:** 10.1038/srep44744

**Published:** 2017-03-17

**Authors:** Rong Zhang, Huan Lu, Yuan-yuan Lyu, Xiao-mei Yang, Lin-yan Zhu, Guang-dong Yang, Peng-cheng Jiang, Yuan Re, Wei-wei Song, Jin-hao Wang, Can-can Zhang, Fei Gu, Tian-jiao Luo, Zhi-yong Wu, Cong-jian Xu

**Affiliations:** 1 Department of Obstetrics and Gynecology, Fengxian Hospital, Southern Medical University, Shanghai 201499, China; 2 Obstetrics and Gynecology Hospital, Fudan University, Shanghai 200011, China; 3 State Key Laboratory of Oncogenes and Related Genes, Shanghai Cancer Institute, Renji Hospital, Shanghai Jiao Tong University School of Medicine, Shanghai 200240, China; 4 Department of Obstetrics and Gynecology, Changzhou NO. 2 People’s Hospital, Jiangsu 213003, China; 5 Changzhou Maternal And Child Health Care Hospital, Jiangsu 213003, China; 6 Jinzhou Medical University, Liaoning 121001, China; 7 Department of Obstetrics and Gynecology of Shanghai Medical College, Fudan University, Shanghai 200032, China; 8 Shanghai Key Laboratory of Female Reproductive Endocrine Related Diseases, Shanghai 200011, China; 9 Cancer Institute, Fudan University Shanghai Cancer Center, Shanghai 200032, China; 10 Institutes of Biomedical Sciences, Fudan University, Shanghai 200032, China

## Abstract

Cervical cancer is an infectious cancer and the most common gynecologic cancer worldwide. E6/E7, the early genes of the high-risk mucosal human papillomavirus type, play key roles in the carcinogenic process of cervical cancer. However, little was known about its roles in modulating tumor microenvironment, particular extracellular matrix (ECM). In this study, we found that E6/E7 could regulate multiple ECM proteins, especially collagen triple helix repeat containing 1 (CTHRC1). CTHRC1 is highly expressed in cervical cancer tissue and serum and closely correlated with clinicopathological parameters. CTHRC1 promotes cervical cancer cell migration and invasion *in vitro* and metastasis *in vivo.* E6/E7 regulates the expression of CTHRC1 in cervical cancer by E6/E7-p53-POU2F1 (POU class 2 homeobox 1) axis. Futhermore, CTHRC1 activates Wnt/PCP signaling pathway. Take together, E6/E7-p53-POU2F1-CTHRC1 axis promotes cervical cancer cell invasion and metastasis and may act as a potential therapeutic target for interventions against cervical cancer invasion and metastasis.

Cervical cancer is an infectious cancer and the most common gynecologic cancer worldwide. Repeated and persistent high risk HPV infection is found to be a major cause of cervical cancer and cervical intraepithelial neoplasia (CIN) III^1^,^2^. Extensive studies indicate that the products of the early genes, E6 and E7, of the high-risk mucosal HPV types play a key role in the carcinogenic process of cervical cancer^3^. The viral E6 and E7 oncoproteins inactivate the p53 and pRb proteins and abolish their cancer prevention effects, respectively. However, only minority persistent HPV infection transforms into cervical cancer, so only HPV infection is not enough for malignant transformation of cervical cancer. Additional pathogenic factors, like microenvironment, must be required for cervical cancer development.

Evidence has been accumulated over the past 10 years that the microenvironment in which cancer arises plays a critical role in tumorigenesis^4^,^5^. Tumor microenvironment is a complex integrated system, which play important roles in tumor proliferation, invasion, metastasis, angiogenesis and immune response. The growth and metastasis of tumor depend not only on the cancer cell itself, but also on the interaction between cancer cells and their microenvironment. Extracellular matrix protein is one of the main components of the tumor microenvironment. Induced by either tumour cells or tumour stromal components, matricellular proteins initiate downstream signalling events that lead to proliferation, invasion, matrix remodelling and dissemination to pre-metastatic niches in other organs^6^. However, little research has been performed on how HPV E6/E7 affects microenvironment, particular extracellular matrix proteins and their receptors, and finally leads to the occurrence and metastasis of cervical cancer.

In this study, by silencing of E6/E7 in high-risk HPV16 or HPV18 infected cervical cancer, we identified multiple microenvironment genes which were altered after E6/E7 silencing. Among them, CTHRC1 is of particular interest to us. Our results showed that CTHRC1 promotes cervical cancer cell migration and invasion *in vitro* and metastasis *in vivo*. We also demonstrated the molecular mechanism of how E6/E7 regulates the expression of CTHRC1, which provides a new evidence for E6/E7 promoting cervical cancer metastasis by modulating microenvironmental factors.

## Results

### Extracellular matrix expression profile is altered by silencing of E6/E7

**Figure 1:**
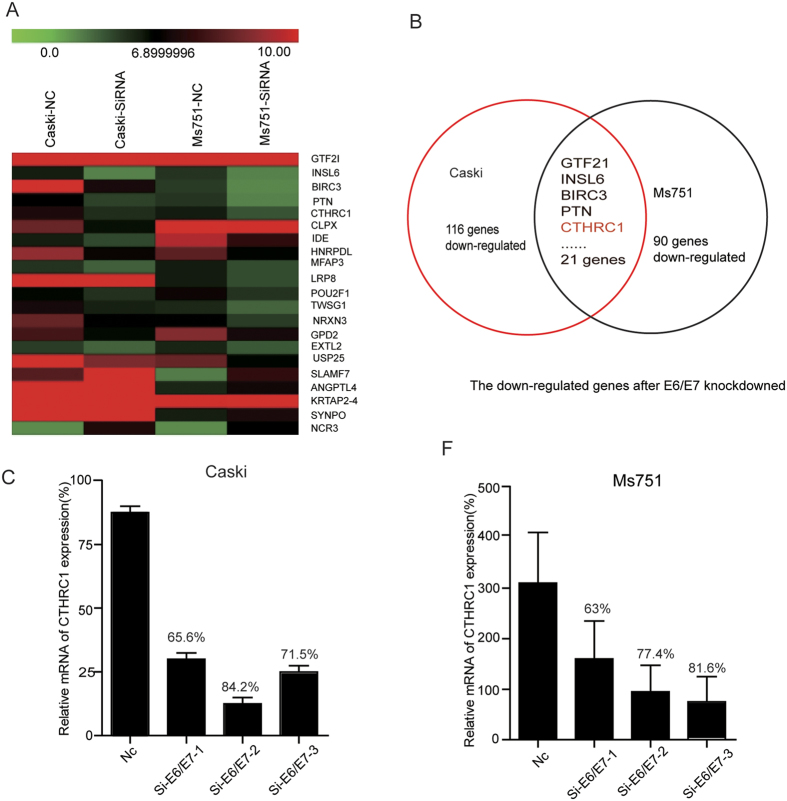
Extracellular matrix expression profile is altered by silencing of E6/E7. (**A**) 21 genes that encode extracellular secreted proteins were significantly down-regulated in Caski and Ms751 cells with silencing of E6/E7 as compared with control cells (fold change >3 and P < 0.05). (**B**) Shows a schematic diagram of down-regulated extracellular secreted proteins. (**C**) The expression of CTHRC1 in Caski cells with silencing of E6/E7, detected by RT-PCR and normalized with 18S expression. (**D**) The expression of CTHRC1 in Ms751cells with silencing of E6/E7, detected by RT-PCR and normalized with 18S expression.

Since E6/E7 play a key role in the carcinogenic process of cervical cancer, we performed a whole-genome microarray analysis by silencing of E6/E7 in Caski and Ms751 cells (Supplementary Figure 1). The results showed that 116 genes were down-regulated and 122 genes were up-regulated in Caski cells, 90 genes were down-regulated and 100 genes were up-regulated in Ms751 cells (fold change >3, *P* < 0.05) (Supplementary Figure 2). Twenty-one extracellular matrix genes including CTHRC1, GTF2I, INSL6, BIRC3, PTN, CLPX, IDE, HNRPDL, MFAP3, LRP8, POU2F1, TWSG1, NRXN3, GPD2, EXTL2, USP25, SLAMF7, ANGPTL4, KRTAP2-4, SYNPO and NCR3 were down-regulated in both cells (Fig. 1A and B). Among them CTHRC1 is of particular interest to us. The qPCR result confirmed that the expression of CTHRC1 was greatly reduced in Caski cells and Ms751 cells after silencing of E6/E7 (Fig. 1C and D).

### CTHRC1 is highly expressed in cervical cancer and closely correlated with clinicopathological parameters

**Figure 2:**
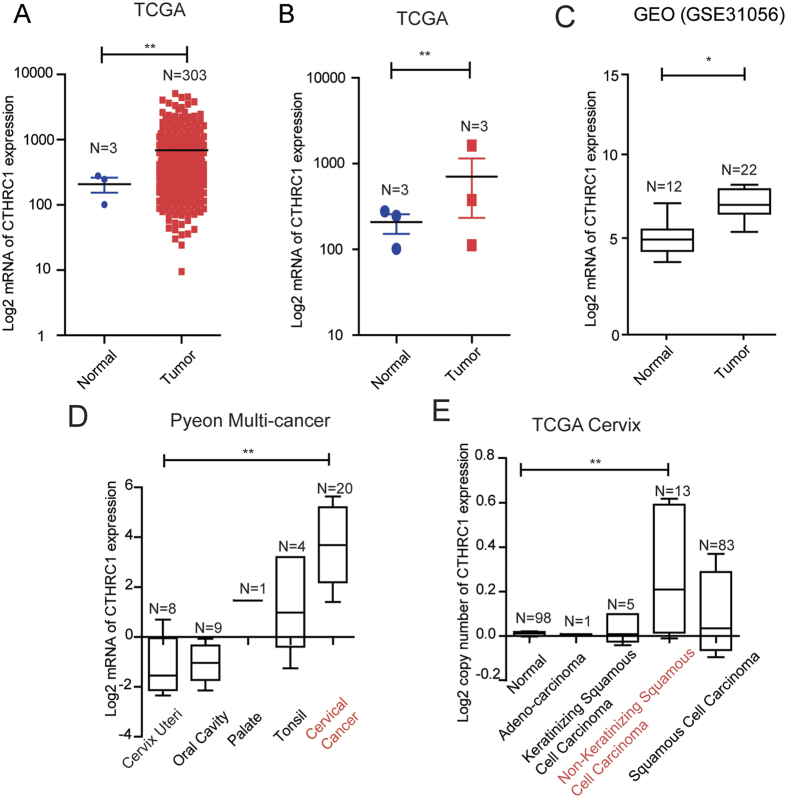
CTHRC1 expression was significantly up-regulated in cervical cancers. (**A**) The mRNA expression of CTHRC1 is upregulated in tumor tissues compared with the normal tissues revealed using the TCGA dataset. (**B**) The mRNA expression of CTHRC1 is upregulated in three matched tumor and non-tumor tissue revealed using the TCGA dataset. (**C**) CTHRC1 expression in the normal and tumor tissues revealed by the GSE31056 dataset. (**D**) The mRNA expression of CTHRC1 was analyzed in different cancers in Oncomine datasete about 42 women. (**E**) The copy number of CTHRC1 expression in different tissues was analyzed in TCGA dataset about 200 women.

**Figure 3:**
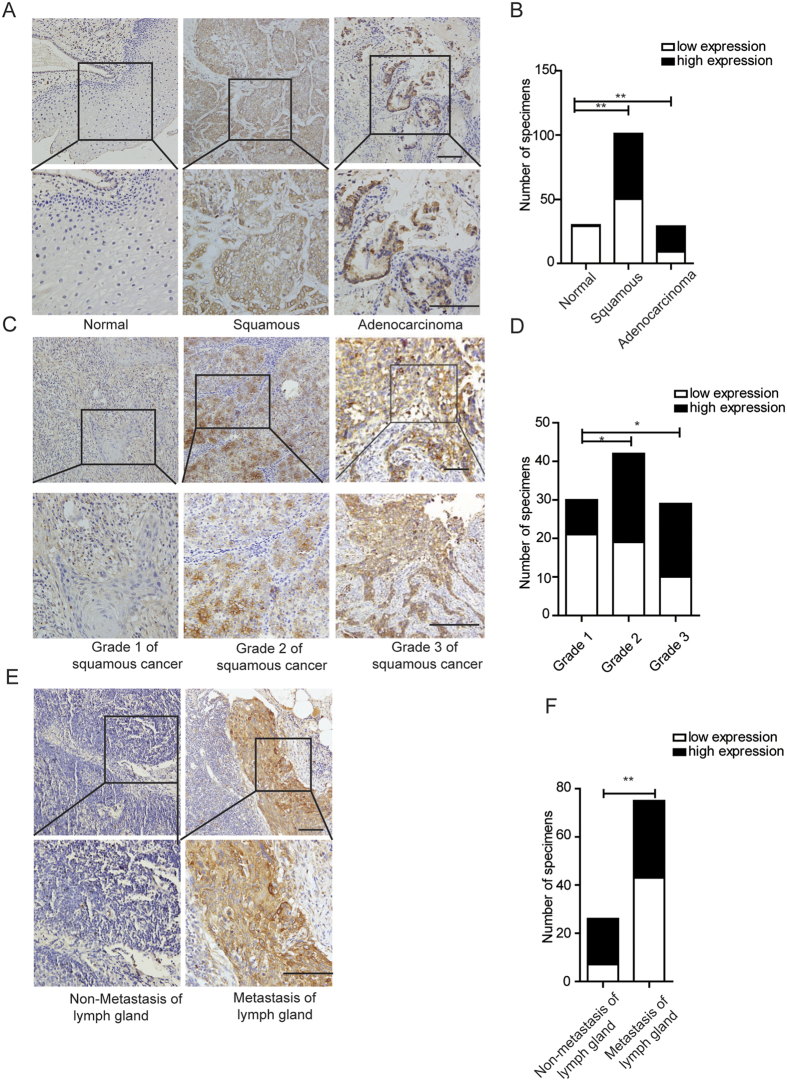
Immunohistochemical analysis of CTHRC1 in cervical cancer tissues. (**A**) Representative photographs of the CTHRC1 immunoreactivity in Normal, Adenocarcinoma, and Squamous cancer tissues. (**B**) Comparisons of CTHRC1 expression in tissues revealed by IHC analysis in Normal, Adenocarcinoma, and Squamous cancer tissues. (**C**) Representative photographs of the CTHRC1 staining in grade 1, 2,3 squamous cervical carcinoma tissues. (**D**) Comparisons of CTHRC1 expression in tissues revealed by IHC analysis grade 1, 2,3 squamous cervical carcinoma tissues. (**E**) Representative photographs of the CTHRC1 staining in Non-metastasis and metastasis of lymph gland tissues. (**F**) Comparisons of CTHRC1 expression in tissues revealed by IHC analysis Non-metastasis metastasis of lymph gland tissues. The positive staining of CTHRC1 is shown in brown color, and the cell nuclei were counterstained with hematoxylin. Scale bars, 10 μm. Original magnification: 200 × . Data are means ± SD. *P < 0.05, **P < 0.01.

To further explore the clinical significance of CTHRC1, we firstly analyzed the expression of CTHRC1 in cervical cancer using The Cancer Genome Atlas (TCGA), Gene Expression Omnibus (GEO) dataset and Oncomine (http://www.oncomine.org). The expression of CTHRC1 in 303 cervical cancer tissues was significantly higher than those in normal cervical tissues in TCGA (Fig. 2A). In another TCGA dataset, the expression of CTHRC1 also increased in 3 cervical tumor tissues compared to the matched non-tumor tissues (Fig. 2B). In a dataset from GEO (GSE31056), the expression of CTHRC1 in 22 cervical cancer tissues increased compared to 12 normal tissues (Fig. 2C). Moreover, the expression of CTHRC1 in 20 cervical cancer was higher than those in cervix uteri (n = 8), oral cavity (n = 9), palate (n = 1) and tonsil (n = 4) in Oncomine dataset (Fig. 2D). Higher copy number of CTHRC1 was especially found in 13 non-keratinizing squamous cell carcinoma as compared with normal (n = 98), adeno-carcinoma (n = 1), keratinizing squamous cell carcinoma (n = 5) and squamous cell carcinoma (n = 83) in TCGA cervix (Fig. 2E). Further, we performed an immunohistochemical analysis of CTHRC1 in a tissue microarray containing 101 cervical squamous cell carcinoma tissue samples, 29 cervical adenocarcinoma tissue samples, 19 cervical intraepithelial neoplasia (CIN) and 30 normal cervical tissues. 130 cervical cancer tissue samples were obtained from patients infected with HPV16/18. Stronger CTHRC1 staining was detected in the cervical squamous cell carcinoma tissues and cervical adenocarcinoma tissues than that in CIN and normal cervical tissues (Fig. 3A and B). To further investigate the clinical significance of CTHRC1 expression in cervical cancer, we examined the correlation between the CTHRC1 expression status and clinicopathological characteristics of 101 cervical squamous cell carcinoma patients who were divided into two groups: the high expression group (n = 51) and the low expression group (n = 50). The results indicated that the expression level of CTHRC1 was closely associated with clinical stages (*P* = 0.0021), pathology grade (*P* = 0.0186) (Fig. 3C and D), lymph metastasis (*P* = 0.0075) (Fig. 3E and F), Lymphatic vascular invasion (*P* = 0.001), deep of invasion (*P* = 0.0001) and diameter of tumor (*P* < 0.0001) (Table 1).

### CTHRC1 has no effect on cervical cancer cell proliferation *in vitro* and tumor growth *in vivo*

**Figure 4:**
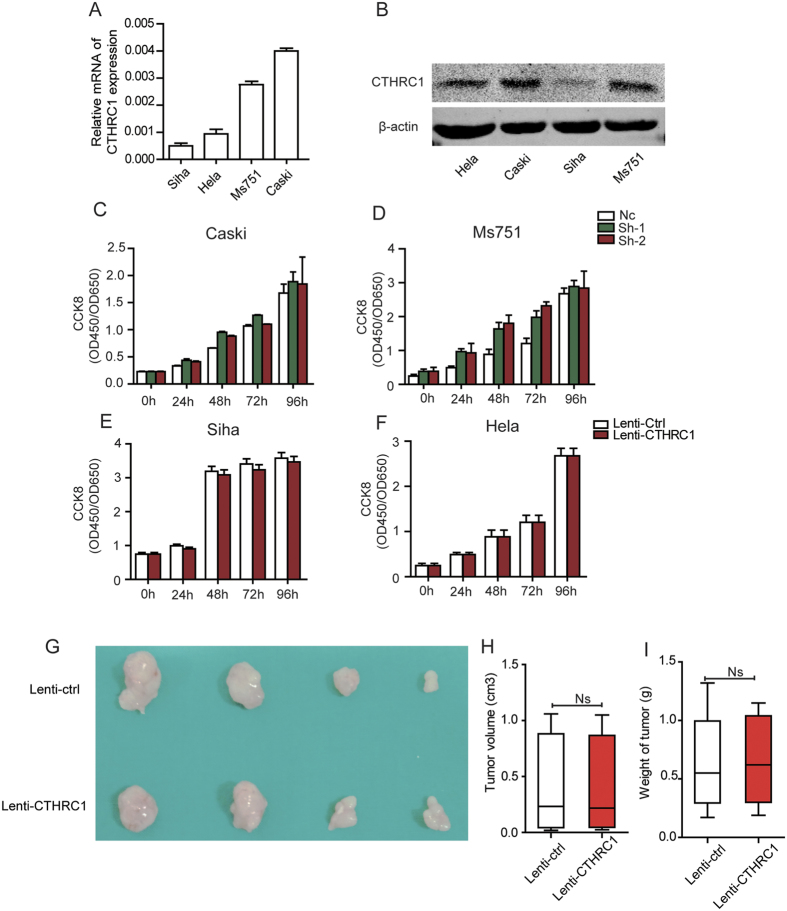
CTHRC1 have no effect on cells proliferation *in vitro* and tumor growth *in vivo*. (**A** and **B**) The expression of CTHRC1 in cell lysates from Caski, Ms751, Siha and Hela cells was detected by RT-PCR and normalized with 18S expression, western blot and normalized with β-actin expression. (**C** and **D**) The cell proliferation of Nc and sh-1, sh-2 groups in Caski (**C**) and Ms751 (**D**) cells were determined by CCK8 assay at 0, 24, 48, 72, 96 h, respectively. Values are means ± SD, n = 5. (**E** and **F**) The cell proliferation of control and Lenti-CTHRC1 in Siha and Hela cells was determined by CCK8 assay at 0, 24, 48, 72, 96 h, respectively. Values are means ± SD, n = 5. (**G**) Morphologic characteristics of tumors from mice inoculated with Siha/Control and Siha/Lenti-CTHRC1 cells. (**H** and **I**) Tumor volumes and tumor weights of Nc and Lenti-CTHRC1 groups from G. n = 6.

To explore the biological functions of CTHRC1 in cervical cancer progression, the expression of CTHRC1 in cervical cancer cells was detected by qPCR and western blotting (Fig. 4A,B and Supplementary Figure 6Aand B). CTHRC1 highly expressed cells Caski and Ms751 were transfected with shRNA-CTHRC1-(1, 2), designated as sh1 and sh2, or a mock vector, which was labeled as Nc. The silencing effects of the Lenti-shRNAs in these two cells were validated by qPCR and western blotting (Supplementary Figures 3A,B and 6C,D). Meanwhile, we established CTHRC1-overexpressing stable cells, which were transfected with a lentivirus carrying the CTHRC1 gene and labeled as Lenti-CTHRC1, using CTHRC1 lowly expressed cell lines Siha and Hela. Control cells were transfected with a mock vector and designated as Lenti-Crtl. CTHRC1 overexpression in these two cervical cancer cells was confirmed by qPCR and western blotting (Supplementary Figures 3C,D and 6E,F).

We first examined the effect of CTHRC1 overexpression/silencing on cervical cancer cell growth. The results showed that the overexpression/silencing of CTHRC1 has no effect on the proliferation of the cervical cancer cells *in vitro* by Cell Counting Kit-8 (CCK8) assay (Fig. 4C–F). To further confirm the results *in vivo*, Lenti-Crtl or Lenti-CTHRC1 cells (Siha) were subcutaneously inoculated into nude mice. The tumors derived from the Lenti-CTHRC1 cells had no difference with those derived from the Lenti- Crtl cells (Fig. 4G). The average volume and weight of Lenti-Crtl mice were 0.32 ± 0.03 cm^3^ and 0.57 ± 0.02 g in contrast to 0.3 ± 0.05 cm^3^ and 0.589 ± 0.03 g in Lenti-CTHRC1 mice (Fig. 4H and I).

### CTHRC1 promotes cervical cancer cell migration and invasion *in vitro* and metastasis *in vivo*

**Figure 5:**
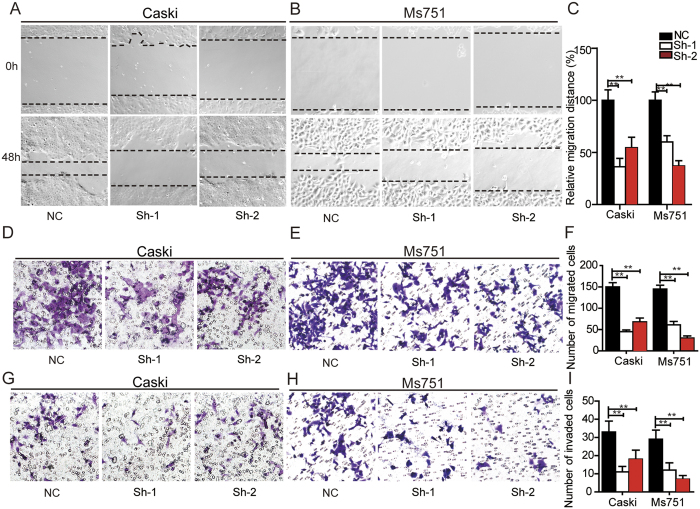
Silencing of CTHRC1 suppresses cervical cancer migration and invasion *in vitro*. (**A** and **B**) Representative wound healing images of Caski (**A**) and Ms751 (**B**) at 0 and 48 h, respectively. The black line outlined the cell boundary. (**C**) Quantification of wound healing rates was analyzed in Caski and Ms751 cells respectively. Data are means ± SD of the wound area relative to the Nc group, n = 3. (**D** and **E**) Representative migration images of CTHRC1 silenced and Nc cells. (**F**) Quantification of migration rates was analyzed in Caski and Ms751cells respectively. (**G** and **H**) Representative invasion images of CTHRC1 silenced and Nc cells. (**I**) Quantification of invasion rates was analyzed in cells. Original magnification: 200 × . Quantifications of cells on the lower surface of the membrane were performed with three randomly selected fields. Data are means ± SD. *P < 0.05, **P < 0.01.

**Figure 6:**
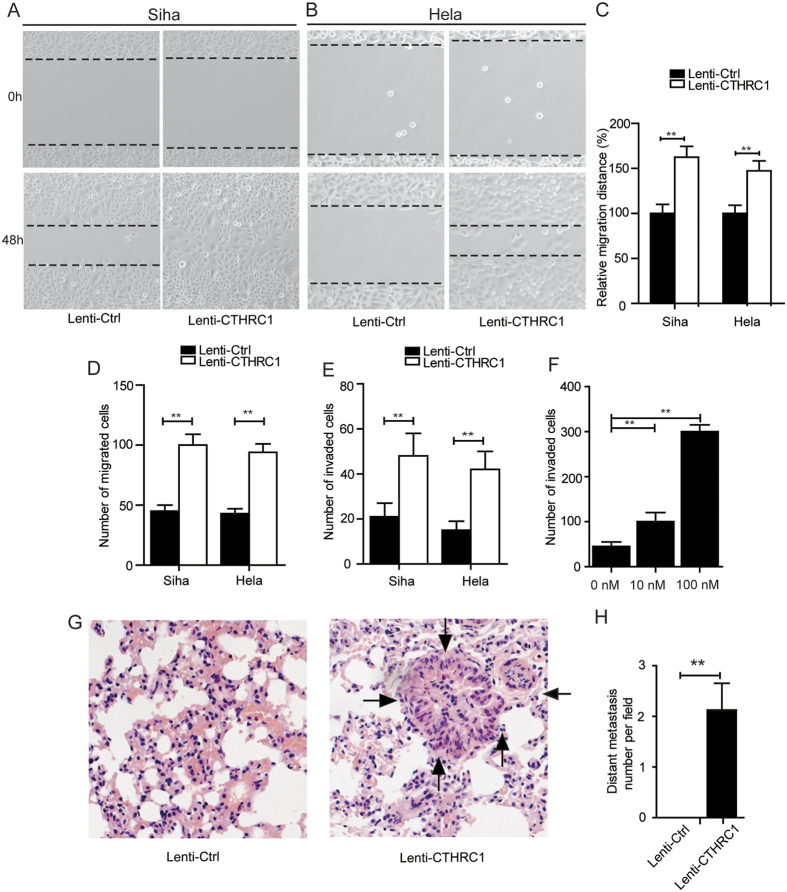
CTHRC1 can promote cervical cancer migration and invasion *in vitro*. (**A** and **B**) Representative wound healing images of Siha (**A**) and Hela (**B**) at 0 and 48 h, respectively. The black line outlined the cell boundary. (**C**) Quantification of wound healing rates was analyzed in Siha and Hela cells respectively. Data are means ± SD of the wound area relative to the control group, n = 3. (**D**) Quantification of migrated rates was analyzed in Siha and Hela cells respectively. (**E**) Quantification of invaded rates was analyzed in Siha and Hela cells respectively. (**F**) Statistical analysis of the cell migrated stimulated by rCTHRC1 protein. (**G**) Pulmonary metastases were detected by H&E staining. (**H**) Statistical analysis of numbers of pulmonary metastatic nodules, n = 3. Original magnification: 200 × . Quantification of cells on the lower surface of the membrane were performed with three randomly selected fields and shown on the right panels. Data are means ± SD. *P < 0.05, **P < 0.01.

We next investigated the effects of CTHRC1 on cervical cancer cell migration and invasion *in vitro*. The results showed that compared to the control group, silencing of CTHRC1 significantly inhibited Caski (Fig. 5A,C,D and F) and Ms751 (Fig. 5B,C,E and F) cell migration *in vitro* by wound healing assay and transwell migration assay. Moreover, silencing of CTHRC1 also suppressed cervical cancer cell invasion *in vitro* by transwell invasion assay (Fig. 5G–I). On the other hand, the overexpression of CTHRC1 significantly promoted Siha and Hela cell migration and invasion *in vitro* by wound healing assay, transwell migration assay and transwell invasion assay (Fig. 6A–E and Supplementary Figure 4A–D). Further, CTHRC1 was recombinantly expressed in 293 T cells, purified and verified by western blotting^7^ (Supplementary Figure 8A). Then, the purified recombinant CTHRC1 (rCTHRC1) protein was applied to primary Siha cells in a transwell migration assay. Compared to the control group, Siha cell migration was significantly enhanced by rCTHRC1 protein at doses of 10 nM and 100 nM. Moreover, the promotion of cell motility by the rCTHRC1 protein was dose-dependent (Fig. 6F and Supplementary Figure 4E).

To examine the role of CTHRC1 in cervical cancer cells metastasis *in vivo*, using a microsyringe, the pulmonary of nude mice were orthotopically inoculated with Lenti-CTHRC1/Siha or Lenti-Crtl/Siha cells. After four weeks, the mice were sacrificed and their pulmonarys were examined. Histological examination of the pulmonary tissue showed that CTHRC1 overexpression promoted the metastatic of cervical cancer *in vivo* (Fig. 6G). The number of pulmonary metastatic nodules was lower in the mice inoculated with the Lenti-Crtl/Siha cells than in the mice inoculated with the Lenti- CTHRC1/Siha cells (Fig. 6H).

### E6/E7-p53- POU2F1 axis regulates CTHRC1 high expression in cervical cancer

Furthermore, we investigated the potential mechanisms of how E6/E7 regulates the expression CTHRC1. It is well-known that E6/E7 oncogenic proteins induces the transformation of epithelial cells, through the disruption of p53 function^8^. So we hypothesized E6/E7 may regulate the expression of CTHRC1 through p53 in cervical cancer. Therefore, we used several on-line tools, like JASPAR (http://jaspar.genereg.net/) and InsulatorDB: (http://insulatordb.utmem.edu) and analyzed whether the promoter region of CTHRC1 has some binding sites for p53. But we did not find any p53 binding sites located in the promoter region of CTHRC1, suggesting p53 may not directly regulate the expression of CTHRC1. Other transcription factors may act as an intermediator between p53 and CTHRC1.

**Figure 7:**
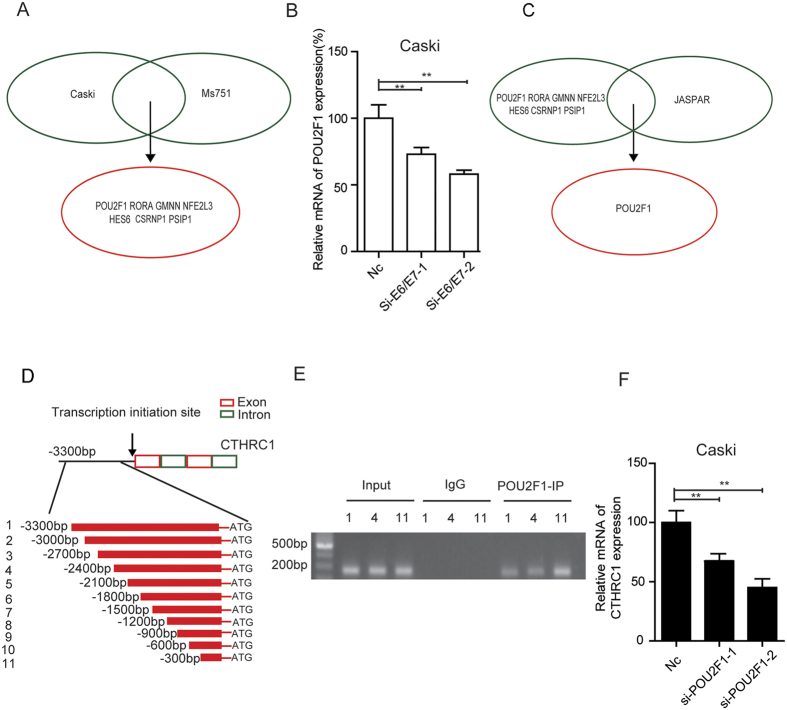
POU2F1 can regulate the exprssion of CTHRC1. (**A**) Seven transcription factors were significantly down-regulated in Caski and Ms751 cells with silencing of E6/E7 as compared with control cells (fold change > 3 and P < 0.05). (**B**) Silencing of E6/E7 in Caski significantly decreased POU2F1 expression, detected by RT-PCR and normalized with 18S expression. (**C**) Transcription factor POU2F1 maybe the target gene combined the transcription profiling microarray and online tools. (**D**) Schematic structure of CTHRC1 promoter. The red and green rectangles indicate exon and intron of CTHRC1 respectively. The zone between black lines represent the primers used in ChIP analysis. (**E**) A ChIP assay was performed using chromatin from Caski cells. (**F**) The expression of CTHRC1 was decreased with silencing of POU2F1 in Caski cells, detected by RT-PCR and normalized with 18S expression.

To find which specific transcription factor directly regulates CTHRC1 expression by silencing of E6/E7, the transcription profiling microarray of Caski and Ms751 after silencing of E6/E7 was further analyzed. The results showed that 7 transcription factors (POU2F1, RORA, GMNN, NFE2L3, HES6, CSRNP1, PSIP1) were significantly downregulated (fold change >3, P < 0.05) (Fig. 7A). However, our qPCR results showed that only the mRNA expression of POU2F1 reduced when knockdown E6/E7 (Fig. 7B and Supplementary Figure 5A). Combining with the analysis of on-line tools, JASPAR and InsulatorDB:, transcription factor POU2F1 may be the direct regulator of CTHRC1 expression (Fig. 7C), which was confirmed by ChIP assay in Caski cells (Supplementary Figure 5B). The results showed that POU2F1 could bind to CTHRC1 promoter at three different regions (Fig. 7D,E and Supplementary Figure 7A). Furthermore, the expression of CTHRC1 was greatly reduced in Caski cells after silencing of POU2F1 (Fig. 7F and Supplementary Figure 5C). Together, these data indicated that transcription factor POU2F1 can directly regulate CTHRC1 expression in cervical cancer cells.

**Figure 8:**
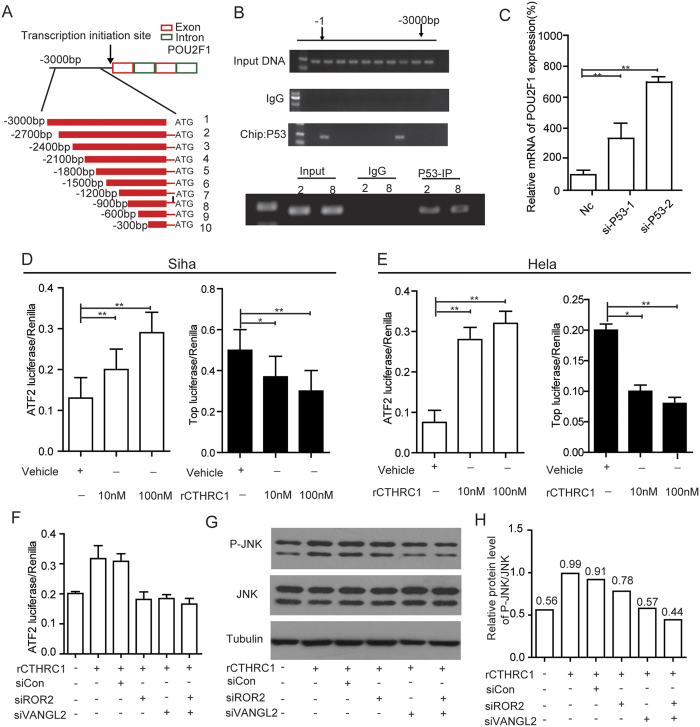
CTHRC1 activates Wnt/PCP signaling pathway. (**A**) Schematic structure of POU2F1 promoter. The red and green rectangles indicate exon and intron of POU2F1 respectively. The zone between black lines represent the primers used in ChIP analysis. (**B**) A ChIP assay was performed using chromatin from Caski cells. (**C**) Silencing of P53 in Caski decreased POU2F1 expression, detected by RT-PCR and normalized with 18S expression. (**D** and **E**) Dual-luciferase reporter assay showed that rCthrc1 protein inhibited Wnt/β-catenin signaling and noncanonical Wnt/PCP was activated in Siha and Hela cells by rCTHRC1 protein in a dose-dependent manner. (**F**) Dual-luciferase reporter assay showed the promotive effect of rCTHRC1 protein on Wnt/PCP signaling was almost blocked after sliencing of ROR2, VANGL2 or ROR2 + VANGL2, respectively. (**G**) Western blot analysis of phosphorylation level of JNK after rCTHRC1 (100 nM) treatment in Hela cells. Treatment of HeLa cells with rCTHRC1 could promote the phosphorylation of JNK, while sliencing of ROR2, VANGL2 or ROR2 + VANGL2, respectively, almost fully reversed this phenomenon. (**H**) Quantitative analysis of grey value for phospho-JNK/total JNK ratio using ImageJ software.

We further asked whether p53 can directly regulate the expression of POU2F1. Analyzed by JASPAR and InsulatorDB:, we found the promoter region of POU2F1 do have the binding sites for p53. ChIP assay further confirmed that p53 can directly bind to the promoter region of POU2F1 in cervical cancer cells (Fig. 8A,B, Supplementary Figures 5E and 7B). Moreover, the expression of POU2F1 was increased after silencing of p53 in Caski cells (Fig. 8C and Supplementary Figure 5D). Together, these data indicated that CTHRC1 expression was directly regulated by E6/E7-p53- POU2F1 axis in cervical cancer.

### CTHRC1 activates Wnt/PCP signaling pathway

It has been reported that CTHRC1 selectively activated Wnt PCP signaling by stabilizing the Wnt-receptor complex^9^. Here, we examined whether CTHRC1 can affect the canonical Wnt pathway or the non-canonical Wnt pathway in cervical cancer cells. A Wnt/β-catenin reporter plasmid (TCF/catenin plasmid) and Wnt/PCP reporter plasmid (ATF2 plasmid) were transfected into cervical cancer cells, Siha and Hela. After transfection with the plasmids for 24 h, recombinant CTHRC1 or vehicle control was added and luciferase activity was determined. The results showed that Wnt/β-catenin signaling was inhibited while the non-canonical Wnt/PCP signaling was activated by rCTHRC1 protein in Siha and Hela cells. Moreover, the activation/inhibition of Wnt signaling by the rCTHRC1 protein was dose-dependent (Fig. 8D and E). Then we examined the effect of CTHRC1 on the JNK activation after knockdown the Wnt/PCP pathway-specific coreceptors ROR2 and VANGL2. The results showed that phosphorylation levels of JNK were increased when treated with rCTHRC1 at doses of 100 nM. Moreover, phosphorylation levels of JNK were decreased after knockdown of ROR2, VANGL2 or ROR2 + VANGL2, respectively, compared to that of the control group (Fig. 8G,H and Supplementary Figure 8B,C). ATF2 luciferase reporter assay showed the promoting effect of rCTHRC1 protein on Wnt/PCP signaling was almost blocked by sliencing of ROR2, VANGL2 or ROR2 + VANGL2, respectively (Fig. 8F).

### CTHRC1 is highly expressed in the serum of cervical cancer patients

**Figure 9:**
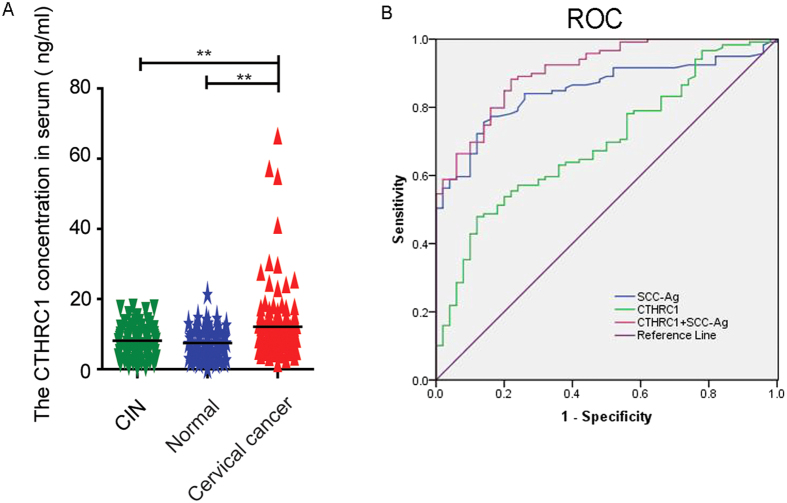
CTHRC1 is highly expressed in the serum of cervical cancer patients. (**A**) Comparisons of CTHRC1 concentration in serum of Cervical intraepithelial neoplasia (CIN), Normal, cervical cancer patients. (**B**) ROC curves of CTHRC1, SCC-Ag and combined. The results shown are mean ± SD of relative firefly/Renilla ratio.

Secreted proteins are ideal candidates of diagnostic and prognostic markers, which can be easily detected in the serum of patients. As a secreted protein, we determined the diagnostic value of CTHRC1 for cervical cancer patients. We measured the serum levels of CTHRC1 in 72 healthy people, 74 CIN patients and 119 cervical cancer patients. The results showed the serum CTHRC1 levels of cervical cancer patients, CIN patients and healthy people are 12.1 ± 0.89 ng/ml, 8.157 ± 0.5100 ng/ml and 7.525 ± 0.4762 ng/ml, respectively. The concentrations of CTHRC1 in serum of cervical cancer were significantly higher than those in CIN patients and healthy people. The difference between healthy controls and CIN patients was not significant (Fig. 9A). As serum squamous carcinoma antigen (SCC-Ag) value is a clinical-used diagnostic marker for cervical cancer, we drew ROC curve of CTHRC1, SCC-Ag and combined CTHRC1 and SCC-Ag to assess the value of CTHRC1 as a serum marker for cervical cancer. For diagnosing cervical cancer, the overall classification accuracy of CTHRC1 was 69.4%, with 48.7% sensitivity and 86% specificity (Table 2). A multivariate function combining measurement of serum concentrations of SCC-Ag and CTHRC1 improved overall sensitivity for detection of cervical cancer to 88.2%. Combining measurement of these two markers can improve the AUC (area under roc curve) to 0.909 ± 0.022. (Fig. 9B).

## Discussion

Although there has been an effective early screening tool for cervical cancer, cervical cancer is still one of the most common cancers among women of reproductive age in low- and middle-income countries^10^. Moreover, using traditional treatment, the 5 years survival rate of cervical cancer patients is relatively low, due to cancer invasion and metastasis^10^. Tumor microenvironment, especially extracellular matrix proteins, an important components of tumor microenvironment, play a crucial role in tumor invasion and metastasis. Interactions between extracellular matrix proteins and their receptors initiate downstream signaling pathways leading to tumor invasion and metastasis^11^. At present, the mechanism of extracellular matrix proteins in cervical cancer microenvironment remain largely elusive^12^. CTHRC1, an extracellular matrix protein, was identified in a screen for differentially expressed sequences in balloon-injured versus normal arteries^13^. CTHRC1 expression is elevated in many human solid tumors, such as melanoma^14^, non small cell lung cancer^15^, colorectal cancer^16^ and gastric carcinoma^17^, and is associated with cancer tissue invasion and metastasis. This study showed that

CTHRC1 is highly expressed in cervical cancer and promotes cervical cancer cell migration and invasion *in vitro* and metastasis *in vivo.* CTHRC1, an extracellular matrix protein, is secreted by cervical cancer cells and acts in a paracrine manner for regulating stromal cells in cancer microenvironment, eg. immune cells, fibroblast and endothelial cells, to influence the invasion and metastasis of cervical cancer.

HPV16 and HPV18 are two of the most important high-risk types of HPV associated with cervical cancer, and about 70% of cervical cancer cases are associated with HPV16 and HPV 18^18^. The E6 and E7 oncoproteins interfere with cell cycle regulators and induce genomic instability, which results in a malignant phenotype^19^. In addition to cervical cancer, E6/E7 is also associated with other tumors. It has been reported that HPV E6-induced promoter hypermethylation of the XRCC3 and XRCC5 DNA repair genes contributes to lung tumorigenesis in nonsmokers^20^. HPV-16 E6 promotes tumorigenesis in esophageal cancer via down-regulation of miR-125b^21^. However, little is known about how E6/E7 promote the development of cancer by remodeling the microenvironment. In this study, we silenced E6/E7 in Caski and Ms751 cell and the whole-genome microarray analysis showed that many tumor microenvironment associated genes were down-regulated, such as NCR3, a gene associated with immune response, and BIRC3, a gene related to regulation of inflammatory response. It suggests that E6/E7 may be closely related to many aspects of tumor microenvironment.

Many studies have demonstrated that the interaction between tumor cells and their microenvironment plays an important role in the process of tumorigenesis^22^. Tumor microenvironment is a complex integrated system, which is different from the microenvironment formed from normal cells with their surrounding tissues. Tumor associated fibroblasts have a profound influence on the development and progression of carcinomas^23^. The immune system has the capacity to promote carcinogenesis, tumor progression, and metastasis^24^. The crosstalk between malignant and nonmalignant cells *via* cytokines and chemokines plays a major role in the various steps of breast cancer progression^25^,^26^,^27^. Many studies have reported that the tumor microenvironment is the key factor for the invasion and metastasis of cervical cancer^28^,^29^,^30^. It is well-known that HPV E6/E7 is a key factor in the pathogenesis of cervical cancer. The major role of E6 is to mediate the degradation of p53 and bind to PDZ-domains on DLG (discs large) and hDLG (Drosophila large) tumor suppressor genes. The primary function of the E7 protein is to inactivate members of the pRb family of tumor suppressor proteins. However, its relationship with the tumor microenvironment is rarely reported. In this study, by silencing E6/E7 in HPV16 and HPV18 infected cervical cancer cells, we found that E6/E7 can regulate many microenvironment factors, like CTHRC1. Further detailed mechanism studies showed that CTHRC1 expression was finely regulated by E6/E7-p53- POU2F1 axis in cervical cancer.

Our study explored the relationship between E6/E7 and the extracellular matrix proteins in the tumor microenvironment, which extends a new theoretical basis for E6/E7 is the key to cervical cancer. However, there are still some limitations in our research. Many microenvironmental factors are significantly altered by silencing of E6/E7, but we only studied the function of CTHRC1 in cervical cancer. In addition, CTHRC1 may have other functions in tumor microenvironment, like microenvironment remodeling or immune response, etc.

In the future, based on this study, we will further explore the relationship between E6/E7 and other factors in the tumor microenvironment, especially uncovering the underlying molecular mechanisms that E6/E7 promotes cervical tumor progression by suppressing the immune response.

## Methods and Materials

### Clinical samples

Human cervical tissue microarray contained 101 cervical squamous cell carcinoma tissue samples, 29 cervical adenocarcinoma tissue samples, 19 cervical intraepithelial neoplasia (CIN) and 30 normal cervical tissues. These tissues were obtained from the Department of Gynecology, Changzhou Maternal and Child Care Hospital and the Department of Obstetrics and Gynecology, Fengxian Hospital, Southern Medical University. None of them had received chemotherapy, radiotherapy and other related anti-tumor therapies before surgery. The institutional ethics committee of the Southern Medical University approved the study protocol, and written informed consent was obtained from participants. The human experiments were performed in accordance with the relevant guidelines, including any relevant details.

### Cell Proliferation Assay, Migration Assay, Invasion Assay, and *In Vivo* Tumor Formation Assay

The details for cell proliferation assay, migration assay and invasion assay are described in the Supporting Information. All animal experiments were approved by the Ethics Review Board of the Southern Medical University and conducted following institutional guidelines.

### Whole-genome microarray construction and Transcriptional profiling microarray

A whole-genome microarray of silencing E6/E7-group and control group in Caski and Ms751 cell lines were done in Shanghai BoHao Biotechnology Co. Ltd (Hao Biotechnology Co, Shanghai, China). A transcriptional profiling microarray of silencing E6/E7 group and control group in Caski and Ms751 cell lines were done in Shanghai BoHao Biotechnology Co. Ltd (Hao Biotechnology Co, Shanghai, China). The details are described in the Supporting Information.

### CTHRC1 Recombinant Protein Expression, Purification and Verification

CTHRC1 was recombinantly expressed in 293 T cells, and further purified and verified by western blotting. The details are described in the Supporting Information.

### Transcriptional reporter gene assay

A Wnt/β-catenin reporter plasmid (TCF/catenin plasmid) and Wnt/PCP reporter plasmid (ATF2 plasmid) were transfected into cervical cancer cells, Siha and Hela. The details are described in the Supporting Information.

The details for immunohistochemistry, cell culture, real-time polymerase chain reaction (PCR), western blotting, lentivirus production and transduction, CTHRC1 recombinant protein expression, purification and verification, and luciferase reporter assays are described in the Supporting Information.

### Statistical analyses

Statistical analyses were conducted using SPSS 21.0 software (Chicago, IL, USA). We performed chi-squared tests in cross tables to assess the relationships between expression levels of CTHRC1 and clinicopathological factors. All statistical tests were two-sided. One-way analysis of variance (ANOVA, Post-hoc testing) was used to compare groups. P value less than 0.05 was considered statistically significant.

## Additional Information

**How to cite this article**: Zhang, R. *et al*. E6/E7-P53-POU2F1-CTHRC1 axis promotes cervical cancer metastasis and activates Wnt/PCP pathway. *Sci. Rep.* **7**, 44744; doi: 10.1038/srep44744 (2017).

**Publisher's note:** Springer Nature remains neutral with regard to jurisdictional claims in published maps and institutional affiliations.

## Supplementary Material

Supplementary Information

## Figures and Tables

**Table 1:** The relationship between the expression of CTHRC1 and clinicopathologic feature of cervical cancer.

Item	Grade	Low expression n = 50	High expression n = 51	Total	*Χ*^2^	*P*-value
Clinical stages	Stage 1	47	36	83	9.4489	0.0021
	Stage 2 and 3	3	15	18		
Pathology grade	Grade 1	21	9	30	7.9649	0.0186
	Grade 2	19	23	42		
	Grade 3	10	19	29		
Lymph metastasis	(+)	7	19	26	7.1426	0.0075
	(−)	43	32	75		
Lymphatic vascular invasion	(+)	9	25	34	10.8788	0.0010
	(−)	41	26	67		
Deep of invasion	>1/2	27	45	72	14.457	0.0001
	≤1/2	23	6	29		
Diameter of tumor	≤4 cm	38	16	54	20.2104	<0.0001
	>4 cm	12	35	47		
Age (years)	≤45	21	19	40	0.2377	0.6259
	>45	29	32	61		

**Table 2:** ROC related parameters for CTHRC1, SCC-Ag and CTHRC1 combined SCC-Ag.

	Cut off	AUC	sensitivity	specificity	Youden
CTHRC1	10.411	0.694 ± 0.042	48.7%	86%	0.347
SCC-Ag	1.495	0.850 ± 0.029	75.6%	86%	0.616
CTHRC1 + SCC-Ag	0.508	0.909 ± 0.022	88.2%	78%	0.662
