# Supplementary material for: E6/E7-P53-POU2F1-CTHRC1 axis promotes cervical cancer metastasis and activates Wnt/PCP pathway
Source: Sci Rep. 2017 Mar 17;7:44744. doi: 10.1038/srep44744 (PMC5356195; doi:10.1038/srep44744)

E6/E7-P53-POU2F1-CTHRC1 axis promotes cervical cancer metastasis and activates Wnt/PCP pathway

Rong Zhang[*1,2], Huan Lu[*1], Yuan-yuan Lyu[*1], Xiao-mei Yang[3], Lin-yan Zhu[1], Guang-dong Yang[1], Peng-cheng Jiang[4], Yuan Re[5], Wei-wei Song[1], Jin-hao Wang[1], Can-can Zhang[6], Fei-Gu[1], Tian-jiao Luo[6], Zhi-yong Wu[#2], Cong-jian Xu[# 2,7,8,9,10]

1.Department of Obstetrics and Gynecology, Fengxian Hospital, Southern Medical University, Shanghai 201499, China; 2.Obstetrics and Gynecology Hospital, Fudan University, Shanghai 200011, China; 3. State Key Laboratory of Oncogenes and Related Genes, Shanghai Cancer Institute, Renji Hospital, Shanghai Jiao Tong University School of Medicine, Shanghai 200240, China; 4.Department of Obstetrics and Gynecology, Changzhou NO. 2 People's Hospital, Jiangsu 213003, China; 5.Changzhou Maternal And Child Health Care Hospital, Jiangsu 213003, China; 6.Jinzhou Medical University, Liaoning 121001, China; 7.Department of Obstetrics and Gynecology of Shanghai Medical College, Fudan University, Shanghai 200032, China; 8.Shanghai Key Laboratory of Female Reproductive Endocrine Related Diseases, Shanghai 200011, China; 9.Cancer Institute, Fudan University Shanghai Cancer Center, Shanghai 200032, China; 10. Institutes of Biomedical Sciences, Fudan University, Shanghai 200032, China.

* *Equal contributors.*

# Correspondence: Zhi-yong Wu, MD, Obstetrics and Gynecology Hospital, Fudan University, Shanghai 200011, P.R. China. Tel: +8602133189900, E-mail: 13918416385@163.com

# Correspondence: Cong-Jian Xu, PhD, Obstetrics and Gynecology Hospital, Fudan University, Shanghai 200011, P.R. China. Tel: +8602163455050, E-mail: xucongjian@yahoo.com

Supporting Information

Cell culture

Caski, Ms751, Hela and Siha cell lines were purchased from the Cell Bank of the Chinese Academy of Science, Ms751, Hela were cultured in DMEM (Hyclone, Beijing, China), Caski and Siha cell lines were cultured in RPMI medium 1640 (Hyclone, Beijing China) supplemented with 10% fetal bovine serum (GBICO, Beijing, China), 100 U/ml penicillin and 100 μg/ml streptomycin. All cells were

maintained at 37 ° C in an incubator with a 5% CO_2 atmosphere.

Whole-genome microarray construction

A whole-genome microarray of silencing E6/E7-group and control group in Caski and Ms751 cell lines were done in Shanghai BoHao Biotechnology Co. Ltd (Hao Biotechnology Co, Shanghai, China). according to manufacturer's protocol. In brief, 1.65 μg Cy3-labeled cRNA from two biological replicates was hybridized to using Gene Expression Hybridization Kit (Cat#5188-5242, Agilent technologies, Santa Clara, CA, US). After 17 hours, slides were washed in staining dishes (Cat#121, Thermo Shandon, Waltham, MA, US) with Gene Expression Wash Buffer Kit. The chip contains 42494 probes , including 5x 301 repeat probes and 2118 internal control probes. The reference database for Probe design is UCSC hg18 (Build NCBI 36, March 2006). The spacing of Probe is 43KB.

Transcriptional profiling microarray

A transcriptional profiling microarray of silencing E6/E7 group and control group in Caski and Ms751 cell lines were done in Shanghai BoHao Biotechnology Co. Ltd (Hao Biotechnology Co, Shanghai, China), according to manufacturer's protocol. In brief, 1.5 μg of labeled cRNA from three biological replicates was hybridized to Illumina Genome-Wide Expression BeadChips (Human Ref-6v.3.0; Illumina Inc.) representing ～43 000 human transcripts at 58 ° C overnight. The array was washed and blocked, and hybridized beads were stained with Streptavidin Cy3 (Sigma, St. Louis, MO, USA). The chips were scanned using Agilent Microarray Scanner (Cat#G2565CA，Agilent technologies, Santa Clara, CA, US).

Immunohistochemical staining

Paraffin embedded tissue samples were cut into 4-μm-thick sections slides, then were stained with primary antibodies for CTHRC1 antibody (mouse monoclonal antibody, 1:100 dilution, HuaAn Biotechnology, Hangzhou, China) overnight at 4° C, All the steps of the immunohistochemical staining were performed according to standard procedures as described. After treatment with

diaminobenzidine and counterstaining with hematoxylin, all the sections were observed and photographed with a microscope (Axio Imager: Carl Zeiss). Scoring was conducted according to the ratio and intensity of positive-staining cells: 0 – 5% scored 0; 6 – 35% scored 1; 36 – 70% scored 2; more than 70% scored 3. The final score of CTHRC1 expression was designated as low or high expression group as follows: low expression: score 0 – 1; high expression: score 2 – 3. All the scores ofCTHRC1 expression were done in a blinded manner and determined independently by two senior pathologists.

CTHRC1 Recombinant Protein Expression, Purification and Verification

CTHRC1 ORF were cloned into the episomal expression vector V152 with p CEP-Pu-Strep II-tag (C-terminal) inframe and the sequence of the CTHRC1 downstream of the CMV promoter. CTHRC1 was recombinant expressed in 293T cells after transfecting reconstructed plasmid by using X-TremeGENE 9 DNA Transfecting Reagent (Roche, Mannheim, Germany). Forty eight hours after transfection, the 293T cells were screening with puromycin (Sigma-Aldrich, St. Louis, MO) at a dose of 2 μ g/ml in DMEM supplemented with 10% FBS for seven days, then the culture media were collected and applied to the Strep Tactin sepharose column(IBA,Gottingen, Germany). After this, the column was washed with binding buffer and eluted by elution buffer containing 2.5 mM desthiobiotin. The collected fractions were further quantified by Nanodrop 2000 spectrophotometer (Thermo Fisher Scientific, Wilmington, DE) and BCA Protein Assay Kit (Pierce. Biotechnology Inc, Rockford, IL) and identified by western blotting assay.

Western Blotting

Cervical cancer cells were lysed in Western and IP lysis buffer (P0013, Beyotime, Jiangsu, China) supplemented with 1mM PMSF (Adamas beta, Shanghai, China). The lysis buffer includes, 20mM Tris (pH7.5), 150mM NaCl, 1%Triton X-100, sodium pyrophosphate, β -glycerophosphate, EDTA, Na3VO4, leupeptin. The All the proteins were separated by SDS-PAGE, followed by blocking in 1% BSA (Bovine Serum Albumin), The NC (Nitrocellulose filter membrane) or PVDF

(Polyvinylidenefluoride) membrane was incubated with antibodies for CTHRC1 (1:1000, mouse, Human, Hangzhou, China) , β -catenin (1:5000, Rabbit, Epitomics), and species-specific secondary antibodies IRDye680 anti-mouse (LI-COR, 1:20,000) and IRDye800 anti-rabbit(LI-COR, 1:10,000). After incubation with the second antibodies, the bounds were revealed by Odyssey imaging system (LI-COR Biosciences, Lincoln, NE). Quantification was analyzed using Image J software.

Cell Viability Assay

Cell viability was detected using a standard Cell CountingKit-8 assay. Control and sh-CTHRC1, Lenti-CTHRC1 cervical cancer cells were seeded into 96-well plates (100 μ l per well) at a density of 2000 cells per well with 100 μ l of complete culture medium. Each group contains five wells. 10 μ l Cell Counting Kit-8 (CCK-8, WST-8, Dojindo, Japan) solution was added to each well after 0 h, 24 h, 48 h, 72 h and 96 h, respectively. In viable cells, CCK8 was metabolized to produce a colorimetric dye that is detected at 450 nm using a microplate reader. The experiment was repeated three times, the optical density was measured using microplate reader at a wavelength of 450nm.

In Vitro Migration and Invasion Assays

For the transwell migration assay, 20000 Control and sh-CTHRC1, Lenti-vector and Lenti-CTHRC1 cervical cancer cells were placed on the top chamber of each insert with the noncoated membrane (Millicell). Cells were trypsinized and resuspended in medium and 700-900 μ l medium supplemented with 10% fetal bovine serum and Siha cells were added rCTHRC1 protein followed gradient doses of 0 nM, 10 nM, 1000 nM respectively were injected into the lower chamber. After 24 hours for Cevical cancer cells in the migration assays, any cells remaining in the top chambers or on the upper membrane of the inserts were carefully removed. After fixation and staining in a dye solution containing 0.1% crystal violet and 20% methanol, cells adhering to the lower membrane of the inserts were counted and imaged through an IX71 inverted microscope

(Olympus Corp. Tokyo, Japan). We carried out invasion assay by adding 100μl matrigel (BD Bioscience, Franklin Lakes, NJ) into top chamber of transwell and placed 8×10^4 Control and sh-CTHRC1,Lenti-CTHRC1 cervical cancer cells onto the matrigel. 48 hours later, the transwell for invasion was ceased and staining.

In vivo tumor xenograft model

Six-week-old female nude (nu/nu) mice (SLAC, Shanghai, China) were injected subcutaneously in the right flank with the stable single cell clones of Siha cells at 5×10^6 infected with Lenti-CTHRC1 or Lenti -Vector in 100μl serum-free medium for each nude mouse, Each group contained 6 mice, the tumor weights were measured and recorded. After 6 weeks, mice were sacrificed, and their tumors were dissected, fixed with phosphate-buffered neutral formalin and prepared for standard histologic examination. Mice were manipulated and housed according to protocols approved by the East China Normal University Animal Care Commission.

In vivo pulmonary metastasis model

The stable single cell clones of Siha cells at 1.5×10^6 infected with Lenti-CTHRC1 or Lenti -Vector, respectively, were suspended in100μl serum-free DMEM/matrigel (1:1) for each nude mouse. These cells were injected intravenously into nude mice (6 in each group, 6 weeks female BALB/c-nu/nu). After 4 weeks, mice were sacrificed, and their lungs were dissected, fixed with phosphate-buffered neutral formalin and prepared for standard histological examination. Mice were manipulated and housed according to protocols approved by the East China Normal University Animal Care Commission. All animals received humane care according to the criteria outlined in the "Guide for the Care and Use of Laboratory Animals" prepared by the National Academy of Sciences and published by the National Institutes of Health.

Transcriptional reporter gene assay

Siha and Hela cells were seeded in 96-well plates and transfected with mixture of

100 ng TCF/catenin reporter plasmid (WNT/β-catenin signaling), or 100 ng ATF2 reporter plasmid (WNT/PCP signaling). After 48 hours of incubation, firefly and Renilla luciferase activities were measured using the dual-luciferase reporter assay system (Promega, Madison, WI) from the cell lysates.

Establishment of stable CTHRC1 cell lines

Short hairpin RNA (shRNA)-containing plasmids were packaged into lenti-virus and virus titers were determined. Two target cell lines, Caski and Ms751, were infected with 1×10^6 recombinant lentivirus-transducing units in the presence of 6 μg/ml polybrene (Sigma, Shanghai, China). The sequences targeting CTHRC1 are as follows;shRNA sequences targeting CTHRC1

Sh1：5’-AUGUGAAAUACCAACGCUGTT-3’; Sh2：5’

-GCUUCUACUGGAUGGAAUUTT-3’

NC: 5’ - AAUUCCAUCCAGUAGAAGCTT-3’,We construct the CTHRC1 over expression plasmids, the full-length construct with CTHRC1 gene was cloned into the PEZ-Lv105 letivirus vector. plasmids were packaged into lenti-virus and virus titers were determined. Two target cell lines, Siha and Hela were infected with the same method. The stable knockdown and over expression cells were selected in the presence of 2μg/ml puromycin. The knockdown efficacy was tested by RT-PCR and western blot.

Quantitative real-time PCR

Total cellular RNA was extracted using Trizol reagent (Takara) and reversely transcribed through Prime Script RT-PCR kit (Takara) according to the protocol. the CTHRC1 mRNA expression was determined by real-time PCR using SYBR Premix Ex Taq (Takara) on a 7500 real-time PCR system (Applied Biosystems) at the following cycling settings: one initial cycle at 95° C for 10 s followed by 40 cycles of 5 s at 95° C and 30 s at 60° C. Data shown are normalized to 18 s expression and represent the average of three repeated experiments. Prime sequences used for E6, E7, CTHRC1,P53,POU2F1,18 s detection are showed in Supplementary Table.

qChIP – PCR Analysis

Caski cells were subjected to ChIP with the Pierce Agarose ChIP Kit (Thermo). Briefly, cells were treated with 37% formaldehyde to crosslink proteins, and terminated with 0.125M glycine. After being performed with sonication, chromatin – protein complexes were immunoprecipitated with 5 mg of anti-POU2F1 antibodies (NB100-91899, Novus), TP53 (SAB1306667, SIGMA) antibodies or 1 mg of mouse IgG. Real-time PCR was performed to amplify the regions of interest or internal negative control regions. The primers used for these studies are listed in Supplementary Table. The fold enrichment ratio was calculated as the value of the ChIP sample versus the corresponding input sample. Samples yielding a twofold enrichment or better were considered positive targets.

sTable. Primer design of Realtime PCR

The name of genes	Primer sequence (5'→3')		Tm
HPV16-E6	Forward Primer	AATGTTTCAGGACCCTACGG	57.8
	Reverse Primer	TCAGGACACAGTGGCTTTTG	57.8
HPV16-E7	Forward Primer	TTTGCAACCAGAGACAACTGA	56.1
	Reverse Primer	GCCCATTAACAGGTCTTCCA	57.8
HPV18-E6	Forward Primer	GCGACCCTACAAGCTACCTG	61.9
	Reverse Primer	GTTGGAGTCGTTCCTGTCGT	59.8
HPV18-E7	Forward Primer	GCATGGACCTAAGGCAACAT	57.8
	Reverse Primer	TGTTGCTTACTGCTGGGATG	57.8
β-actin	Forward Primer	GTGGGGCGCCCCAGGCACCA	60.2
	Reverse Primer	CTCCTTAATGTCACGCACGATTTC	61
CTHRC1	Forward Primer	GCATGCTGTCAGCGTTGGTA	57.8
	Reverse Primer	TCAATGGGAAGAGGTCCTGAA	57.8
GAPDH	Forward Primer	AGCCTCAAGATCATCAGCAATGCC	57.8
	Reverse Primer	TGTGGTCATGAGTCCTTCCACGAT	57.8
POU2F1	Forward Primer	ATGAACAATCCGTCAGAAACCAG	60.2
	Reverse Primer	GATGGAGATGTCCAAGGAAAGC	61
ROR2	Forward Primer	CCTTTCAGAGTTGGAATCGG	59.7
	Reverse Primer	GCTTCTGCTCTCAGTGTCCC	60.1
VANGL2	Forward Primer	TGCGAGGGCCTCTTCATCT	62.7
	Reverse Primer	CAGCACCATAAGCAGGGCA	62.4

Figure legends

Supplementary Figure1

(A and B) The expression of E6, E7 in Caski cells with silencing of E6/E7, detected by RT-PCR and normalized with 18S expression. (D and E) The expression of E6, E7 in Ms751cells with silencing of E6/E7, detected by RT-PCR and normalized with 18S expression.

Supplementary Figure 2

(A) 116 genes that encode extracellular secreted proteins were significantly down-regulated and 122 genes were significantly up-regulated in Caski cells with silencing of E6/E7 as compared with control cells. (B) 90 genes that encode extracellular secreted proteins were significantly down-regulated and 100 genes were significantly up-regulated in Ms751 cells with silencing of E6/E7 as compared with control cells.

Supplementary Figure 3

(A and B) Silencing of CTHRC1 in Caski and Ms751 cells significantly decreased CTHRC1 expression, detected by RT-PCR and normalized with 18S expression, western blot and normalized with β-actin. $P < 0.01$. (C and D) Overexpression of CTHRC1 in Siha and Hela cells significantly increased CTHRC1 expression, detected by RT-PCR and normalized with 18S expression, western blot and normalized with β-actin. Nc: cells transfected with shRNA containing a scrambled sequence; sh-1 and sh-2: cells transfected with two human shRNA plasmids targeting CTHRC1.

Supplementary Figure 4

(A and B) Representative migration images of CTHRC1 Lentied and control cells. (C and D) Representative invasion images of CTHRC1 Lentied and control cells. (E) Treatment with recombinant CTHRC1 protein at doses of 0 nM, 10 nM,100 nM can promote cells migration to the bottom of transwell

filter. Original magnification: 200×. Quantifications of cells on the lower surface of the membrane were performed with three randomly selected fields.

Supplementary Figure 5

(A) The expression of six transcription factors after silencing of E6/E7 in Caski cells, detected by RT-PCR and normalized with 18S expression. (B) POU2F1 expression in Caski, Ms751, Siha and Hela cells revealed by RT-PCR and normalized with 18S expression. (C) The expression of POU2F1 in Caski cells with silencing of POU2F1. (D) The expression of P53 in Caski cells with silencing of P53. (E) P53 expression in Caski, Ms751, Siha and Hela cells revealed by RT-PCR and normalized with 18S expression. Data are means ± SD. $^{**}P < 0.01$.

Supplementary Figure 6

(A and B) The primal graph of CTHRC1 expression in HPV positive cervical cancer cell lines detected by WB and β-actin as control. A is CTHRC1expression and B is the expression of β-actin. (C and D) The primal graph of CTHRC1 expression in Caski and MS751 with transfected by sh-CTHRC1 detected by WB and β-actin as control. C is CTHRC1expression and D is the expression of β-actin. (E and F) The primal graph of CTHRC1 expression in Siha and Hela with transfected by sh-CTHRC1 detected by WB and β-actin as control. E is CTHRC1 expression and F is the expression of β-actin.

Supplementary Figure 7

(A) The primal graph of ChIP assay of CTHRC1 promoter was performed using chromatin from Caski cells. (B) The primal graph of ChIP assay of POU2F1 promoter was performed using chromatin from Caski cells.

Supplementary Figure 8

(A) Verification of affinity purified CTHRC1 protein by Western blotting. (B) The sliencing effect of ROR2

and VANGL2 detected by RT-PCR. (C) The original western blot images of figure 8G.

Supplementary Figure 1

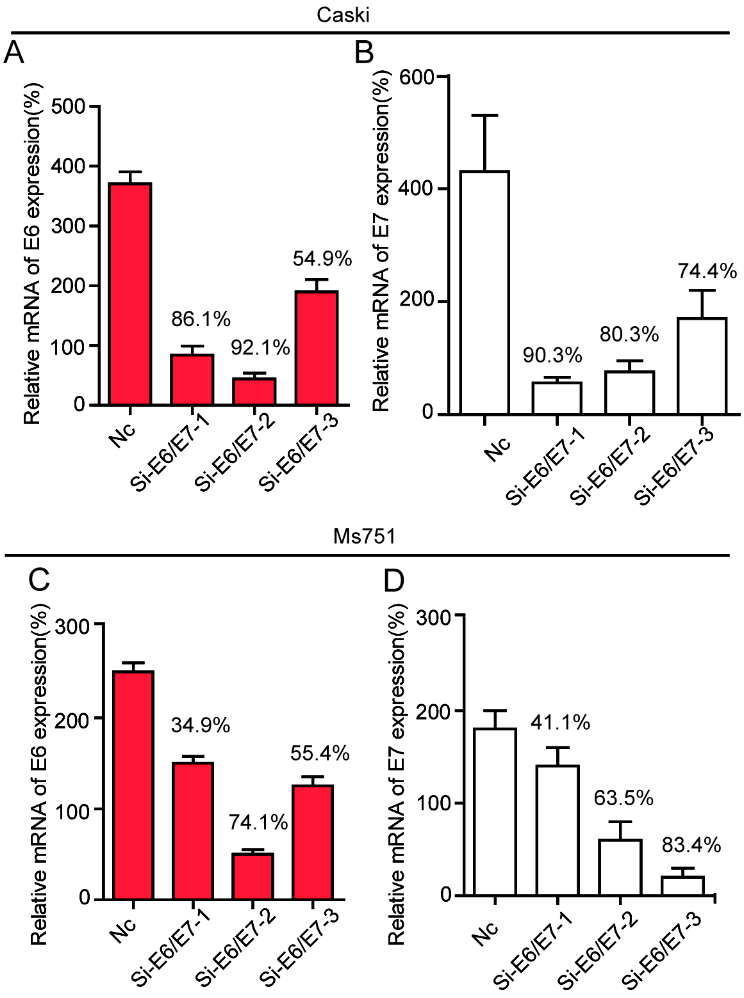

Supplementary Figure 2

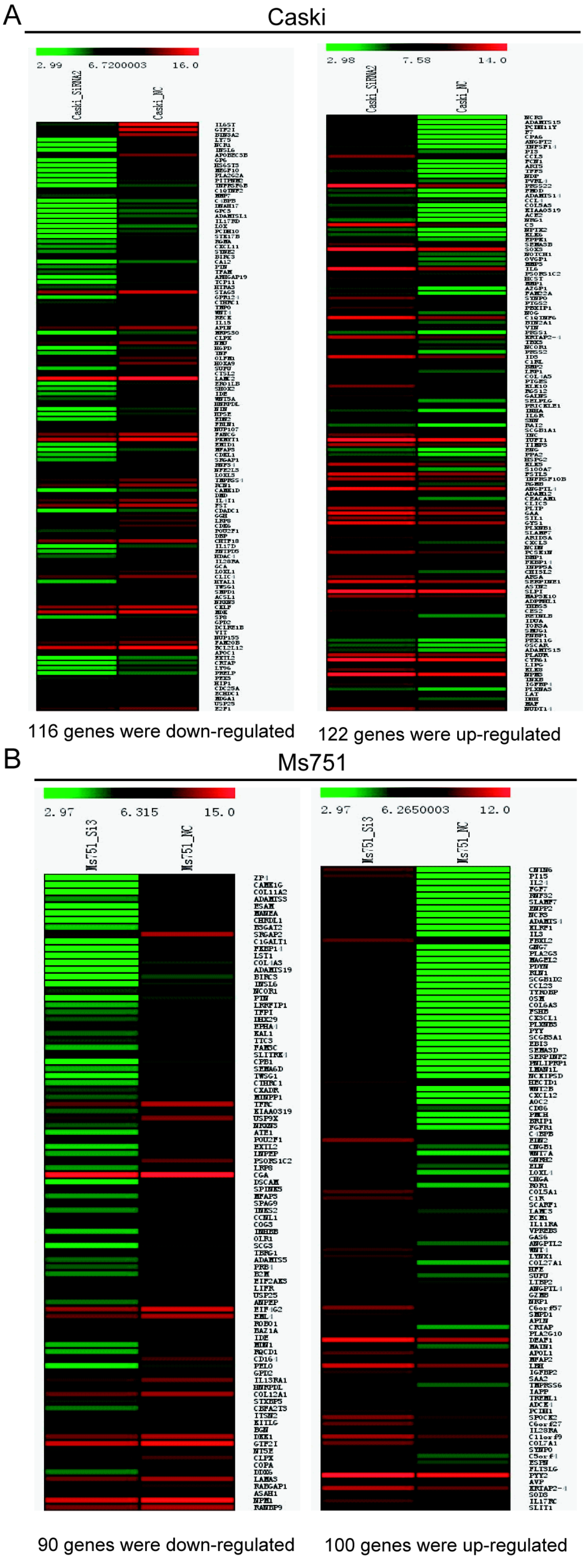

Supplementary Figure 3

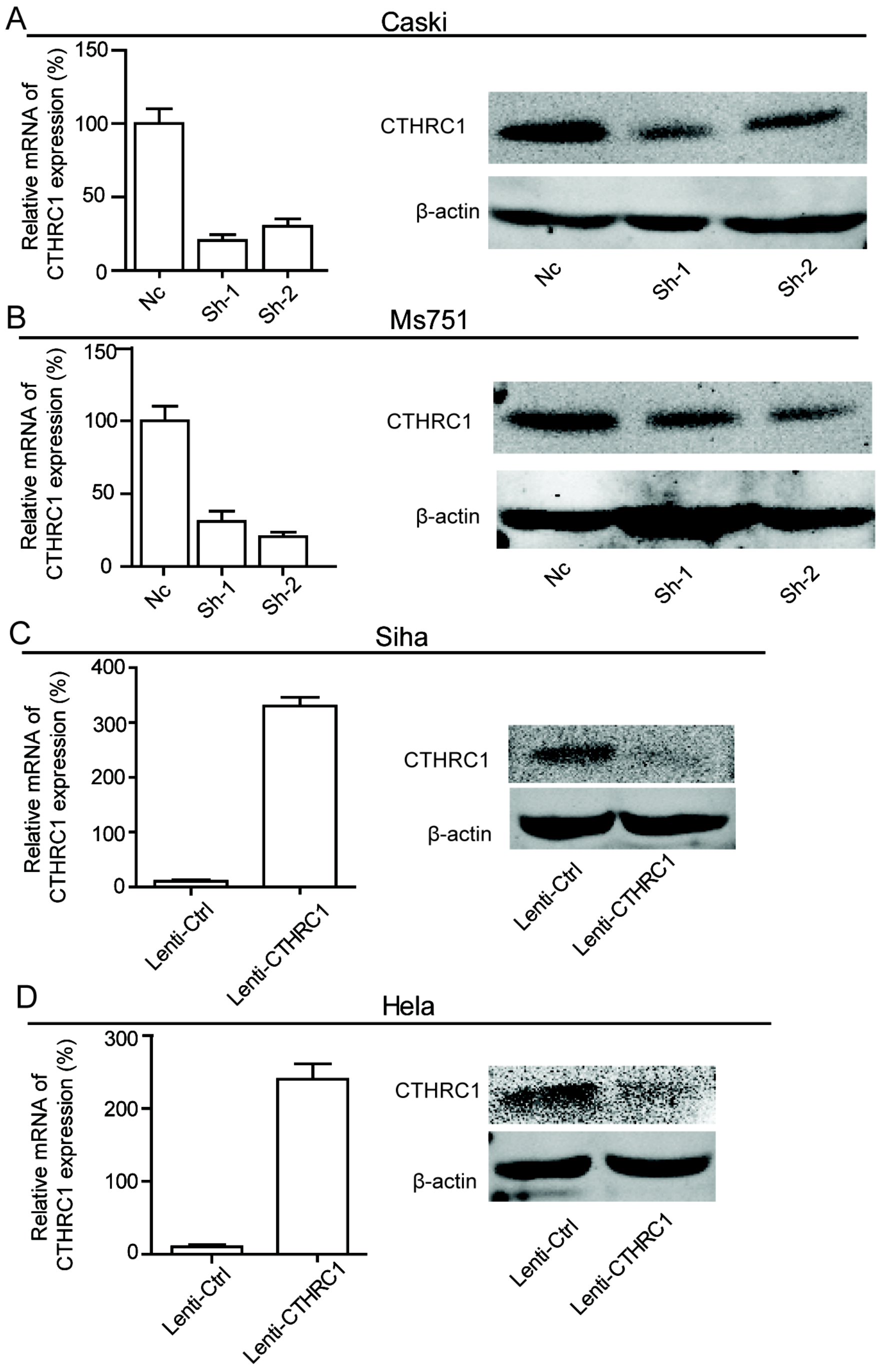

Supplementary Figure 4

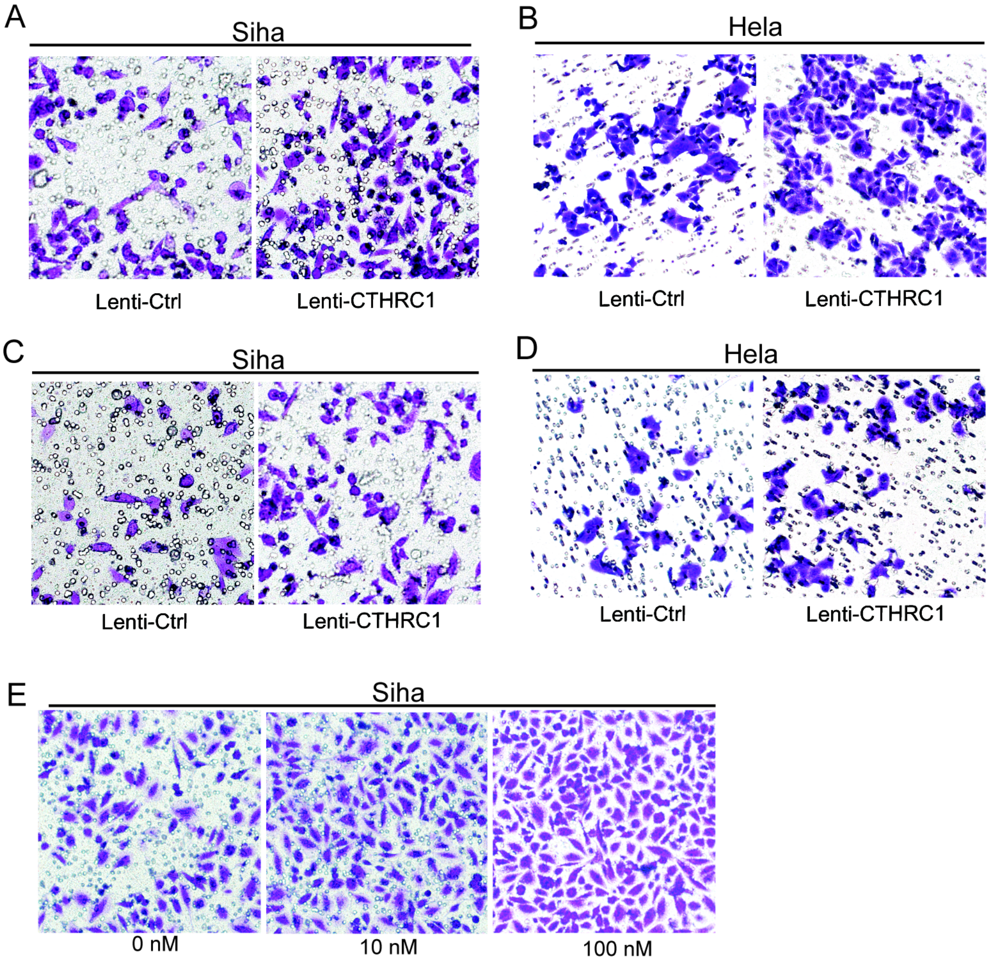

Supplementary Figure 5

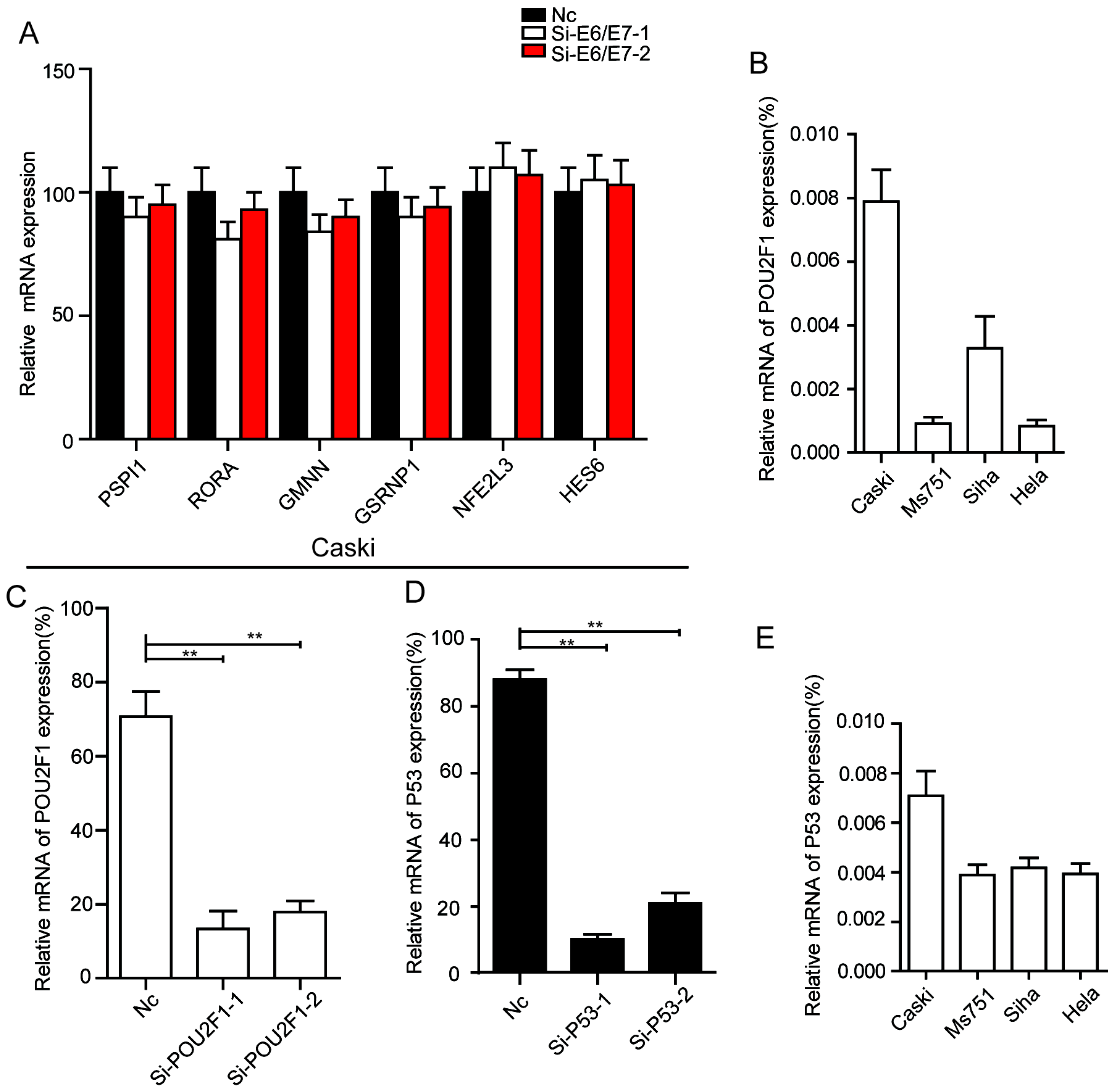

Supplementary Figure 6

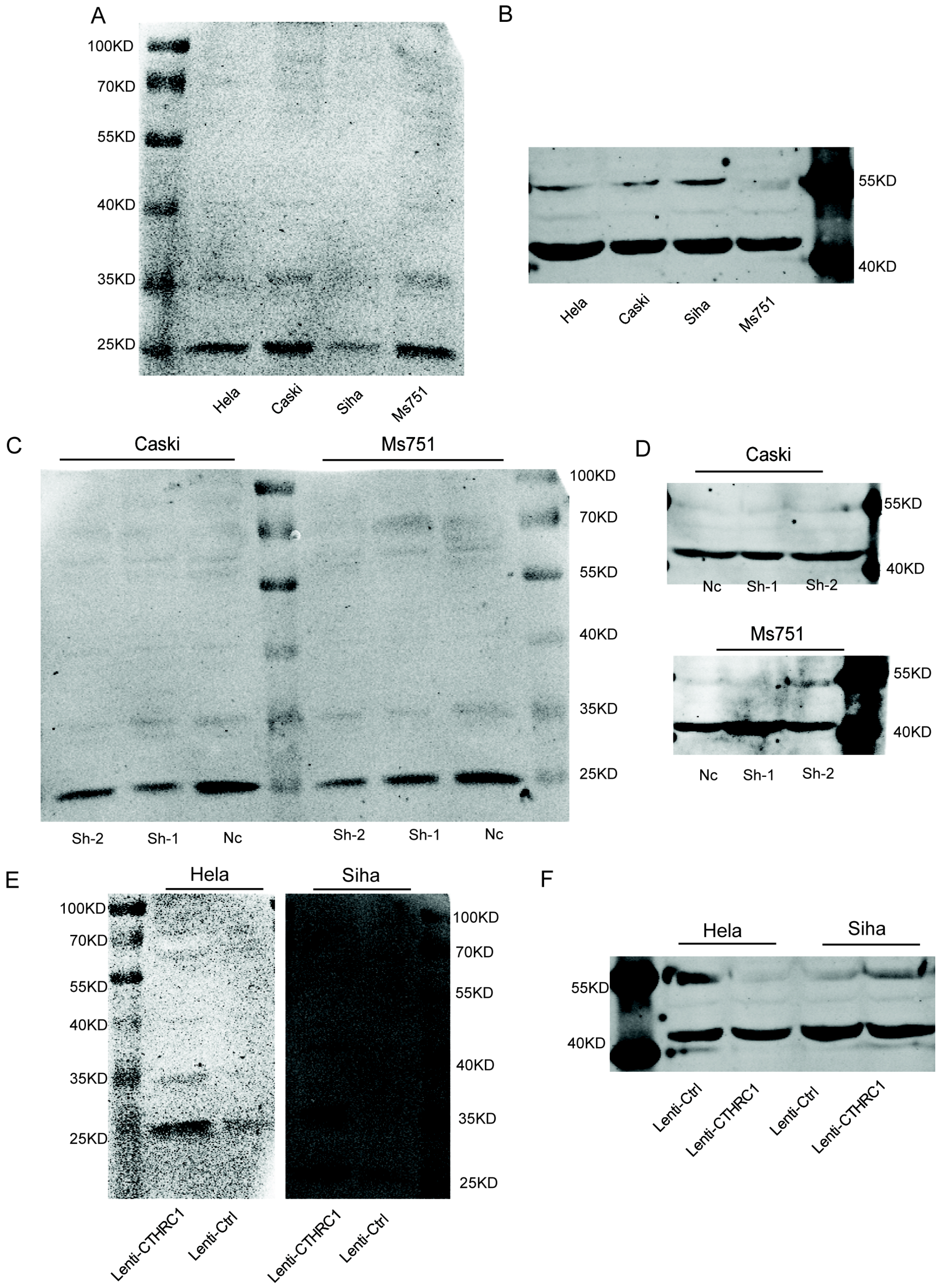

Supplementary Figure 7

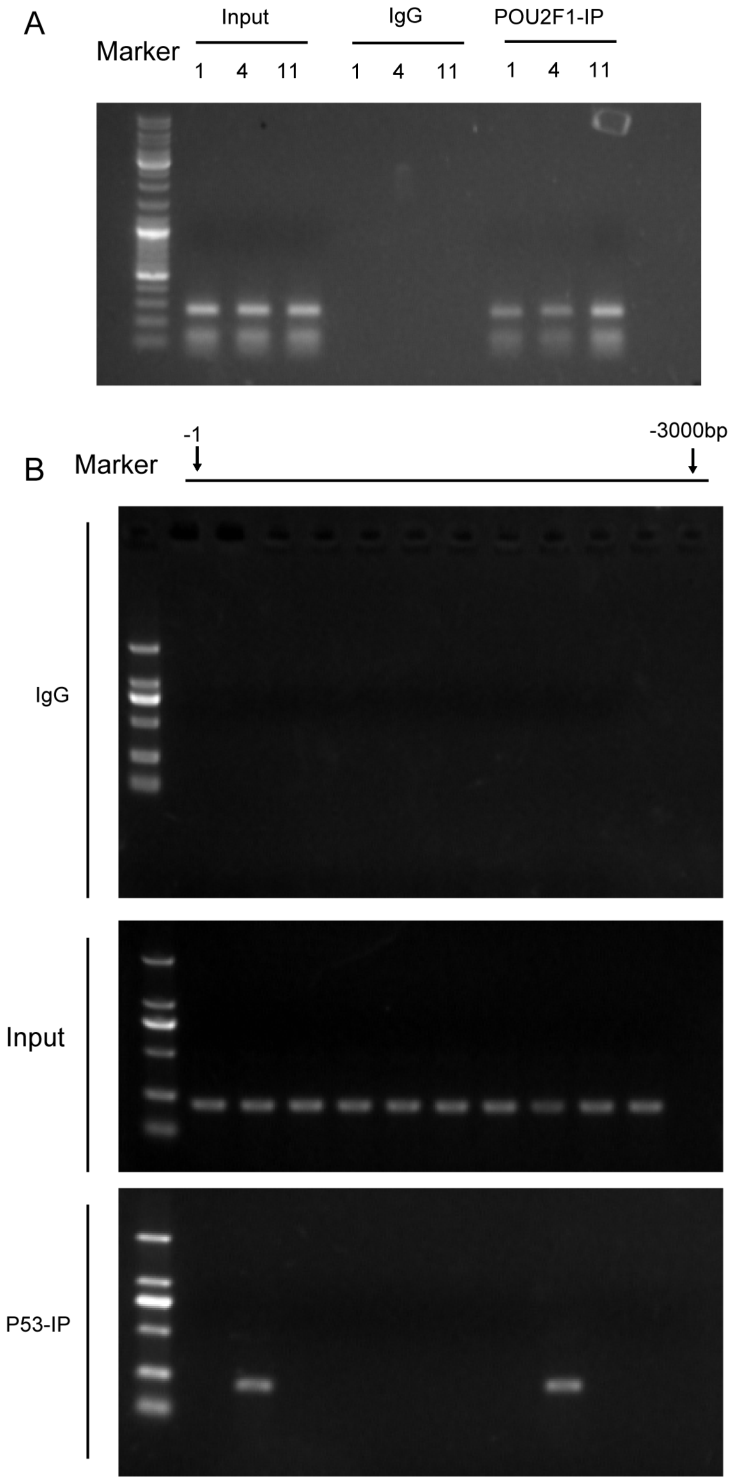

Supplementary Figure 8

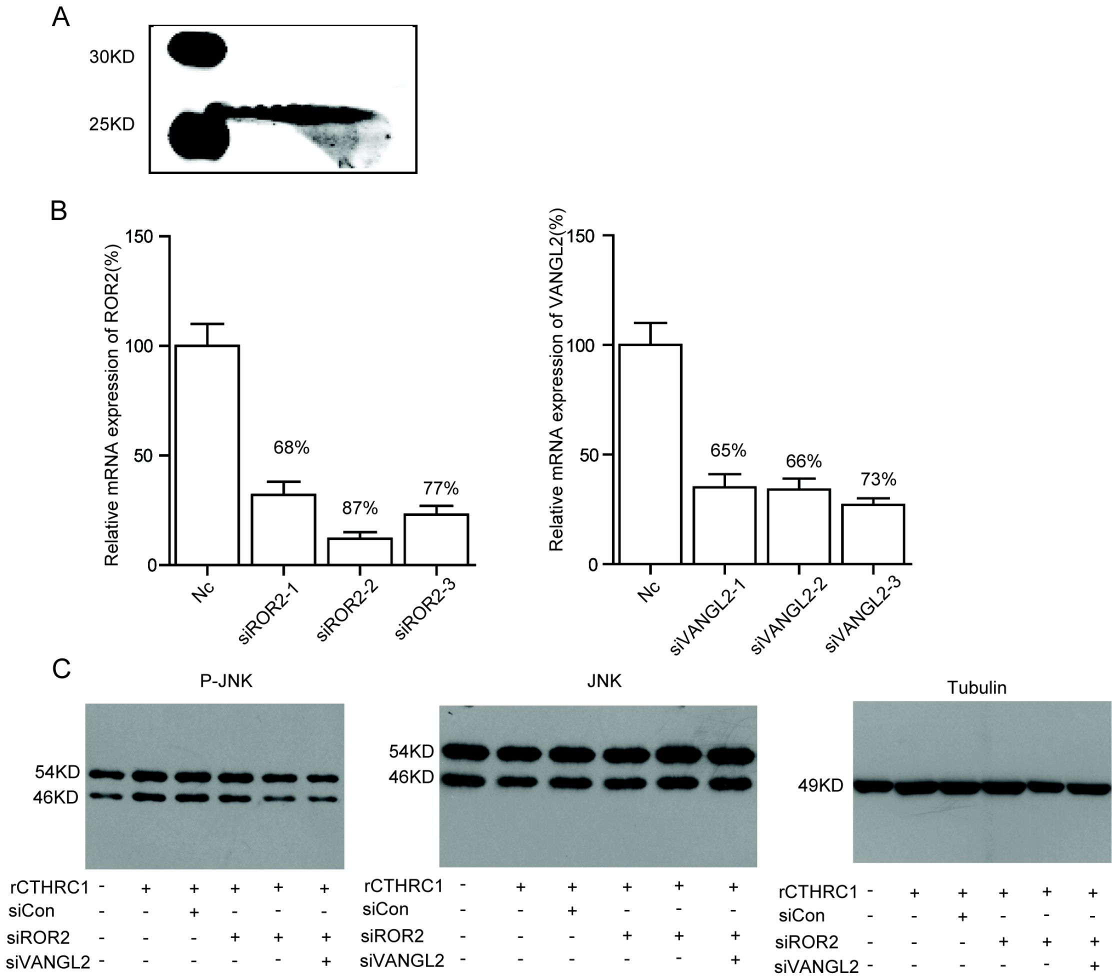

Supplement: Supplementary Information [file srep44744-s1.pdf]
